# Paracolic gutter hematoma after colonic endoscopic submucosal dissection

**DOI:** 10.1093/gastro/goag072

**Published:** 2026-07-15

**Authors:** Guanyi Liu, Shibo Song, Xudong Zhao, Long Rong

**Affiliations:** Endoscopy Center, Peking University First Hospital, Beijing 100034, P. R. China; Endoscopy Center, Peking University First Hospital, Beijing 100034, P. R. China; Endoscopy Center, Peking University First Hospital, Beijing 100034, P. R. China; Endoscopy Center, Peking University First Hospital, Beijing 100034, P. R. China

## Introduction

While endoscopic submucosal dissection (ESD) is a well-established treatment for early colorectal neoplasia, postoperative bleeding remains a known complication, typically manifesting as intraluminal hemorrhage or submucosal hematoma. To our knowledge, however, a paracolic gutter hematoma following colonic ESD has not been previously reported in the literature. This case report describes the clinical presentation, management, and proposed mechanism of this rare complication and discusses technical considerations for its prevention.

## Case report

**Figure 1:**
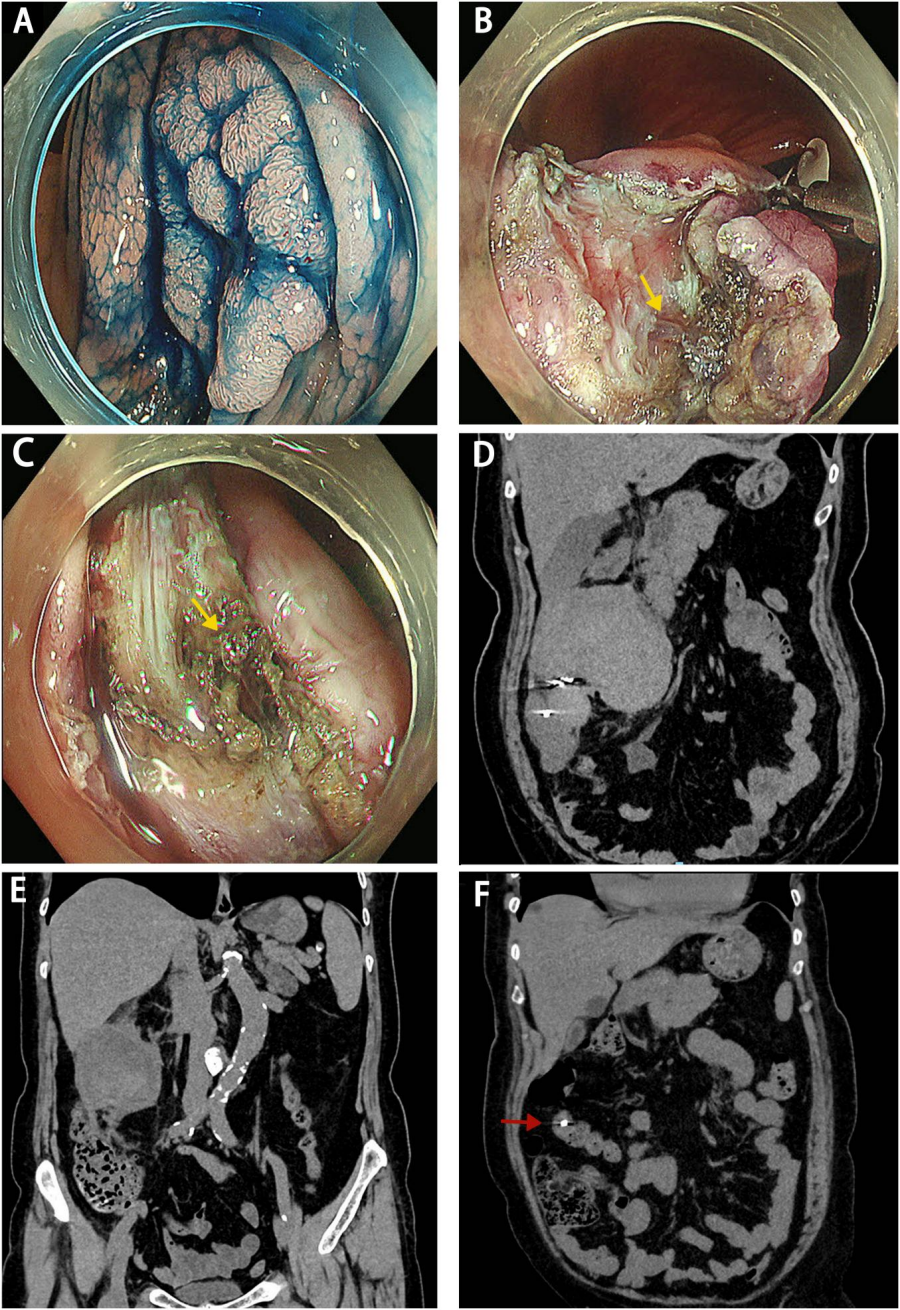
The clinical data of the case with paracolic gutter hematoma after colonic endoscopic submucosal dissection. (A) Indigo carmine chromoendoscopy demonstrated a lateral spreading tumor ∼3 cm in size in the ascending colon. (B) Several perforating vessels originating from the muscularis propria were identified within the central portion of the lesion; the arrow indicates the vessels. (C) After resection, visible vessel stumps could be observed on the ulcer bed, indicated by the arrow. (D) Abdominal CT scan after endoscopic submucosal dissection showed a hematoma in the right paracolic gutter, measuring 11.0 × 6.7 × 11.1 cm^3^; the hyper-dense focus corresponded to the clips at the resection site. (E) One-month follow-up abdominal CT showed a reduction in the size of the right paracolic gutter hematoma to 8.3 × 5.1 × 6.3 cm^3^. (F) Five-month follow-up CT imaging demonstrated complete resolution of the hematoma; the arrow indicates the clips at the resection site.

A 76-year-old woman underwent colonoscopy for constipation. Her past medical history was significant for hypertension, which was well controlled. There was no known history of coagulation disorders, vasculitis, connective tissue diseases or abdominal vascular diseases. She was not on any anticoagulant or antiplatelet medications. A lateral spreading tumor ∼3 cm in size was seen in the ascending colon (Fig. 1A). Biopsy of the lesion suggested high-grade intraepithelial neoplasia. Based on the endoscopic and pathology results, ESD was performed. During the ESD procedure, severe submucosal fibrosis was observed, which hindered the formation of a mucosal flap. To facilitate dissection, internal traction was applied using a clip-with-rubber-band system [1]. Several perforating vessels originating from the muscularis propria were identified within the central portion of the lesion (Fig. 1B). Active bleeding encountered during dissection was effectively controlled using a coagrasper with soft coagulation mode. Following complete resection, visible vessel stumps could be observed on the ulcer bed (Fig. 1C), and the mucosal defect was closed completely with clips. Postoperatively, the patient developed severe abdominal pain. Examination revealed tenderness and rebound tenderness in the right abdomen. Laboratory tests showed a significant elevation in white blood cell count to 19.88 × 10^9^/L and a decrease in hemoglobin of ∼4 g/dL compared to the preoperative level. Despite a marked postoperative drop in hemoglobin, the patient did not present with hematochezia or melena. An abdominal CT scan confirmed the presence of a hematoma in the right paracolic gutter, measuring 11.0 × 6.7 × 11.1 cm^3^ (Fig. 1D). The patient was fasted and managed with fluid resuscitation and antibiotic therapy. We discussed the clinical course with the patient and her family, explaining that if abdominal pain persisted or hemoglobin levels continued to decline, further intervention might be necessary. This could include angiography to localize the bleeding site, followed by transarterial embolization if indicated, or surgical exploration should conservative measures fail. After considering the options, the patient and family decided to continue conservative management and close observation. Following treatment, the patient’s abdominal pain gradually improved. Her white blood cell count returned to normal levels, and the hemoglobin remained stable. She subsequently recovered and was discharged from the hospital. During the follow-up visit 1 month postoperatively, the patient complained of mild abdominal distension. She was given lactulose and probiotics to modulate intestinal motility, maintain regular bowel movements, and relieve symptoms. Follow-up abdominal CT showed a reduction in the size of the right paracolic gutter hematoma to 8.3 ×5.1 × 6.3 cm^3^ (Fig. 1E). By 5 months postoperatively, repeat CT imaging demonstrated complete resolution of the hematoma (Fig. 1F) and the abdominal discomfort resolved.

## Discussion and conclusion

While giant submucosal hematomas following ESD or EMR in the stomach [2–4], and colon [5] have been documented, a paracolic gutter hematoma after colonic ESD has not been previously reported. The intraoperative identification of a large perforating vessel originating from the muscularis propria, coupled with active bleeding during dissection, suggests a possible mechanism for the delayed hematoma. Despite a marked postoperative drop in hemoglobin, the absence of hematochezia or melena indicated that bleeding likely occurred extraluminally. We hypothesize that the bleeding originated from a comparable large vessel which was initially controlled by coagulation during ESD. The coagulation might have been insufficient, and the vessel might have retracted into the muscular layer. Subsequent recanalization could then have led to bleeding that, confined behind the already closed mucosal surface, led to hematoma formation in the paracolic space.

This case highlights a critical technical consideration. During ESD, when encountering a sizable perforating vessel, achieving immediate hemostasis with coagulation should not be considered definitive. It is advisable to apply additional soft coagulation to the vessel stump to ensure complete obliteration before proceeding. Furthermore, during final clip closure of the resection defect, efforts should be made to cover the origin of such perforating vessels whenever feasible. Endoscopists should be alert to this rare complication, which may improve the likelihood of early detection.
